# Platelet Carbonic Anhydrase II, a Forgotten Enzyme, May Be Responsible for Aspirin Resistance

**DOI:** 10.1155/2017/3132063

**Published:** 2017-09-27

**Authors:** M. Jakubowski, J. Dębski, E. Szahidewicz-Krupska, A. Turek-Jakubowska, J. Gawryś, K. Gawryś, R. Skomro, A. Derkacz, A. Doroszko

**Affiliations:** ^1^Department of Internal Medicine, Occupational Diseases and Hypertension, Wroclaw Medical University, Wroclaw, Poland; ^2^Institute of Biochemistry and Biophysics, Polish Academy of Sciences, Warszawa, Poland; ^3^Division of Respiratory, Critical Care and Sleep Medicine, Department of Medicine, University of Saskatchewan, Saskatoon, SK, Canada

## Abstract

**Background:**

Thromboembolic events constitute a major health problem, despite the steadily expanding arsenal of antiplatelet drugs. Hence, there is still a need to optimize the antiplatelet therapy.

**Objectives:**

The aim of our study was to verify a hypothesis that there are no differences in platelet proteome between two groups of healthy people representing different acetylsalicylic acid (aspirin) responses as assessed by the liquid chromatography/mass spectrometry (LC/MS) technique.

**Patients/Methods:**

A total of 61 healthy volunteers were recruited for the study. Physical examination and blood collection were followed by platelet-rich plasma aggregation assays and platelet separation for proteomic LC/MS analysis. Arachidonic acid- (AA-) induced aggregation (in the presence of aspirin) allowed to divide study participants into two groups aspirin-resistant (AR) and aspirin-sensitive (AS) ones. Subsequently, platelet proteome was compared in groups using the LC/MS analysis.

**Results:**

The LC/MS analysis of platelet proteome between groups revealed that out of all identified proteins, the only discriminatory protein, affecting aspirin responsiveness, is platelet carbonic anhydrase II (CA II).

**Conclusions:**

CA II is a platelet function modulator and should be taken into consideration as a cardiovascular event risk factor or therapeutic target.

## 1. Introduction

Despite the steadily expanding arsenal of antiplatelet agents, thromboembolic events are still common in clinical practice [1]. Acetylsalicylic acid (ASA, aspirin), widely used in the high-cardiovascular-risk population, is in many cases ineffective. This phenomenon, called aspirin resistance (AR), remains still an important clinical problem. Its pathogenesis and incidence persist the subject of numerous studies and controversies [2–5]. ASA exerts its anti-aggregatory effect mostly by inhibiting the platelet COX-1 and subsequently thromboxane formation from the arachidonic acid cascade. Aspirin resistance, at molecular level, may be attributed, that is, to changes in efficacy of COX-1 acetylating by ASA [6]. Studies focusing on the AR phenomenon defined in this way do not precise the time frame between the last aspirin dose and blood sampling [6, 7].

On the other hand, ASA treatment failure may result from factors that are COX-1 independent, including noncompliance [7], increased platelet turnover [2], and platelet COX-1 resynthesis [8], and factors increasing platelet reactivity. Up to date, there are several papers describing factors, which expression increases during ASA therapy and which are associated with the AR phenomenon. Studies by Voora et al. show that following 4 weeks of ASA treatment (325 mg/day), the platelet sensitivity to ADP, epinephrine, and collagen significantly increases despite sufficient COX inhibition [9]. The authors introduced a term “aspirin response signature,” which is a set of coexpressed genes assessed in peripheral blood during ASA treatment that are strongly correlated with COX1-independent platelet function and represent increased risk of myocardial infarction and death [10].

Furthermore, Massimi et al. proved that ASA in both *in vitro* cell lines [11, 12] and *in vivo* platelets (over two months of treatment) enhances expression of the multidrug resistance protein 4 (MRP4), high concentration of which is associated with greater TxB2 synthesis, and subsequently more intense collagen-induced aggregation [13]. The platelet MRP4 might be also induced by other nonsteroidal anti-inflammatory drugs [14]. The presence of factor enhancing the “on aspirin” arachidonic acid induced aggregation (exclusively limited to COX-1 pathway) verified by Floyd et al. who have found that truly resistant subjects are characterized by higher platelet expression of glycoprotein IIIa (GPIIIa) following 28 days of ASA treatment (300 mg/d) [15].

Noteworthy, in some studies there is observed interindividual heterogeneity in platelet *in vitro* response to low ASA doses [15]. The question arises if, beside inducible factors limiting ASA chronic treatment, there are constitutively present factors limiting platelet responsiveness to ASA at baseline.

A novel approach—the use of techniques analyzing the functional proteome—should allow to define factors determining individual variability of platelet activity. By analyzing the platelet proteome in patients with ASA resistance, we intended to define novel mechanisms limiting platelet responsiveness to acetylsalicylic acid.

Therefore, the aim of the study was to verify the hypothesis regarding the lack of differences in platelet proteome between groups with different sensitivities to aspirin, as assessed by the liquid chromatography/mass apectrometry (LC/MS) technique.

## 2. Material and Methods

### 2.1. Bioethics Statement

All experiments were conducted and approved in accordance with the guidelines of the local Bioethics Committee and adhered to the principles of the Declaration of Helsinki and Title 45, U.S. Code of Federal Regulations, Part 46, Protection of Human Subjects (revised: November 13, 2001; effective: December 13, 2001). All participants provided their written consent to participate in the study. The written consent forms had been previously approved by the ethics committee.

### 2.2. Recruitment and Examination

A total of 61 clinically healthy volunteers (at the age of 18–60 years) were enrolled to the study. Exclusion criteria were diabetes, hypertension, chronic and acute inflammatory diseases, mental disorders, malignancies, allergy to nonsteroidal anti-inflammatory drugs (NSAID), or the use of NSAID one week prior to the examination.

**Figure 1:**
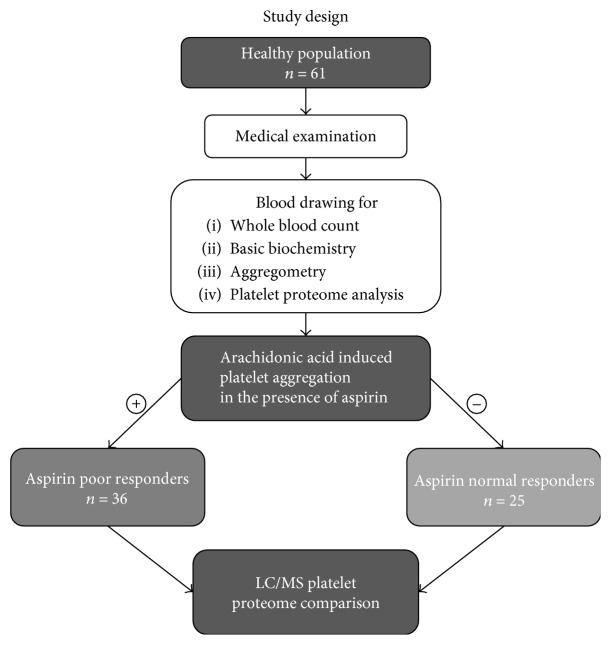
Study protocol.

All participants underwent standard physical examination, and their blood was collected nontraumatically from the antecubital vein using a Sarstedt S-Monovette® system (Sarstedt AG & Co., Nümbrecht, Germany). Study participants were divided into two groups according to the aspirin response (the presence of platelet aggregation in response to arachidonic acid (AA) following an *in vitro* incubation with a fixed dose of ASA) (Figure 1).

At first, baseline characteristics of the groups were compared: then, the presence of differences in the blood test results (including cardiovascular risk factors and markers of endothelial and platelet function) was verified, which was subsequently followed by an analysis of differences in platelet proteome between aspirin-resistant (AR) and aspirin-sensitive (AS) subjects.

### 2.3. Aggregometry

Aggregation was performed in platelet-rich plasma using a 2-channel optical aggregometer (Chrono-log 490-2D, Chrono-Log, Pennsylvania, USA), and platelet response to arachidonic acid with and without aspirin was tested simultaneously. The aggregation discriminating the groups was performed using arachidonic acid (at 1 mM of final concentration) added to platelet-rich plasma (PRP) in 5 minutes following its incubation with acetylsalicylic acid (final concentration 30 *μ*M, for 5 minutes). Since agonist application, the reaction progress was recorded for 6 minutes using dedicated software (Aggro/link, Chrono-Log, Pennsylvania, USA). The aggregation results were calculated according to the manufacturer's instruction by the Chronolog Software and are expressed as the area under the aggregation curve (AUC), which equals zero for no aggregation was calculated during the course of aggregation protocol (from agonist addition until the termination of aggregation at the end of the sixth minute).

A complete inhibition of the aggregation by ASA was the criterion for diagnosing aspirin resistance. The experiment was performed at a constant temperature of 37°C.

Also, control aggregations were performed using other final concentrations of agonist and antagonist: AA 0.5 mM and Lys-ASA 0.03 mM and AA 1 mM and Lys-ASA 0.05 mM. Additionally, in cases when Lys-ASA at 0.05 mM of final concentration was insufficient to prevent the AA-induced aggregation, a test with Lys-ASA at 0.1 mM of final concentration and AA at 1 mM of constant concentration was performed.

### 2.4. Platelet Preparation for Proteomic Analyzes

The whole blood was supplemented with prostacyclin (PGI_2_) at the final concentration of 0.06 *μ*g/ml, and centrifuged for 20 minutes at 230 ×g at 21°C in order to obtain PRP. Subsequently, PRP was supplemented with PGI_2_ (final concentration 0.3 *μ*g/ml) and centrifuged for 10 minutes at 1000 ×g at 21°C. The plasma was discarded, and the platelet pellet was gently washed three times with 1 ml of Tyrodes-HEPES buffer pH 7.4 ml. Rinsed platelets were suspended in 4 ml of Tyrodes-HEPES buffer pH 7.4 supplemented with CaCl_2_ (final concentration 1 mM). Resulted suspension was immediately analyzed for platelet count and contamination with WBC i RBC (Sysmex device, Clinical Laboratories Department, University Hospital, Wrocław, Poland).

The pure PLT suspension was adjusted with Tyrodes-HEPES buffer pH 7.4 containing CaCl_2_ to a final concentration of 2.5 × 10^8^/ml. Samples containing platelets in amounts of 2.5 × 10^8^ and 7.5 × 10^8^ cells were preserved for further proteomic analysis. The samples were obtained by centrifugation of the suspension of known concentration for 5 min, 10000 ×g at 4°C, and stored at −80°C until proteomic analyses.

Aggregations of separated platelets in response to collagen were also performed. A 500 *μ*l of platelet suspension (2.5 × 10^8^/ml) was substituted with collagen (final concentration 5 *μ*g/ml), and aggregation was conducted for 6 min and then immediately stopped by placing cuvettes on ice. Pellet and supernatant were separated by consecutive centrifugation, for 5 min at 10000 ×g and 4°C. Obtained material was stored till proteomic analyses at −80°C.

### 2.5. Liquid Chromatography/Mass Spectrometry (LC/MS)

All used reagents were from Sigma, unless otherwise specified. Platelet proteins were extracted by incubation of platelets in 1% sodium deoxycholate, 10 mM TrisHCl pH 8, with the addition of 0.1% sodium dodecyl sulfate, followed by sonication, and clarified by centrifugation (Eppendorf Minispin, 10 min, 12100 g). Protein concentration was measured, and proteins were reduced with 50 mM phosphine, alkylated with 200 mM thiosulfonate, and digested overnight at 37°C with modified trypsin (V5111, Promega). The digestion reaction was quenched by the addition of 2 *μ*l of 10% trifluoroacetic acid. The concentration of the digested peptides was determined by the Direct Detect Method by Millipore. Sample volume corresponding to 5 *μ*g of digested platelet proteins was subjected for proteomic processing, during which peptides were separated by nano-HPLC C-18 column (nano-ACQUITY Symmetry® BEH C18, Waters 186003545) using an acetonitrile gradient (5–35% in 180 min) in the presence of 0.1% formic acid with a flow rate of 250 nl/min. The chromatographic column outlet was directly coupled to the ESI-LTQ-Orbitrap Velos mass spectrometer (Thermo Electron Corp., San Jose, CA, USA) and was operating in the MS (the measurement of the masses of the peptides) and MS/MS (peptides fragmentation) in data-dependent acquisition. The raw data was processed using Mascot Distiller followed by analysis with Mascot software (Matrix Science), using database Swiss-Prot, with taxonomy restricted to *Homo sapiens* [16]. Peptides with a Mascot score exceeding the threshold value corresponding to <1% FDR were considered to be positively identified. Label-free quantization was performed as described previously [17]. The lists of identified proteins were analyzed using Diffprot software [18].

### 2.6. Endothelial and Platelet Activation Markers

Plasma concentrations of sP-selectin/CD62P and PAI-1 were determined by a sandwich enzyme immunoassay technique, using commercial ELISA kits (Cat: BBE6 and DSE100, R&D Systems Europe Ltd., UK) with a sensitivity of 0.5 ng/ml, according to the manufacturer's instructions. The optical density 450/620 nm was measured with a BioTek Absorbance Microplate Reader with software Gen5. The coefficient of variation (CV) intra-assay %CV was calculated as the ratio of the pooled standard deviation from all samples (each was analyzed in triplicate) and the overall mean and then multiplied by 100. Interassay %CV refers to assay-to-assay consistency that was calculated using the pooled standard deviation divided by the overall mean of all duplicated samples and then multiplied by 100, as previously described [19]. The intra-assay CV was less 6%, and interassay was less 10%.

### 2.7. Measurement of Prostanoids Levels

Plasma concentrations of TxB_2_ and 6-ketoPGF-1alpha were determined using commercial immunoassays by Enzo Life Science, strictly following manufacturer's instructions, as previously described [19].

### 2.8. Statistical Analysis

Data is expressed as the mean ± SEM. The differences between two continuous parameters were assessed using the Mann–Whitney *U* test or Student *t*-test, following the Shapiro-Wilk test and Levene test as appropriate. Proteomics data analysis was performed as described in previous section.

## 3. Results

### 3.1. Baseline Characteristics of Investigated Population in Subgroups

The discriminating aggregation divided the study population into two groups. The aggregation was performed using arachidonic acid (1 mM working concentration) added to platelet-rich plasma in 5 minutes following its incubation with ASA at (30 *μ*M, for 5 minutes). Following the agonist application, the reaction progress was recorded for 6 min. A complete inhibition of the aggregation by ASA was the criterion for distinguishing the AS from AR subjects. The first one constituted 36 subjects whose platelets were resistant to ASA (aspirin resistant (AR)). The second one (age and sex matched) was formed from 25 individuals that presented preserved ASA responsiveness (aspirin sensitive (AS)). The groups' characteristics are shown in Table 1. In a complete blood count, the differences between groups involved only quantitative parameters of red blood cells, hemoglobin level, hematocrit and red blood cell count that were significantly higher in AS. There were no differences observed in the qualitative measurements of erythrocyte line (MCV, MCH, and MCHC).

Regarding biochemical risk factors, significant differences (AR versus AS) concerned only fasting glucose (83.39 ± 1.07 versus 88.52 ± 2.06 mg%, resp., *p* < 0.05) and serum creatinine (0.96 ± 0.02 versus 1.11 ± 0.03 mg%, resp., *p* < 0.05). However, these values were maintained within normal ranges.

### 3.2. Platelet Function

There were no significant differences in platelet count (PLT), size (PDW), and activation level (sP-selectin concentration) between subgroups. Nevertheless, AR subjects were characterized by lower plasminogen activator inhibitor-1 (PAI-1) concentrations (3.1 ± 0.3 versus 3.66 ± 0.3 ng/ml in AS, resp., *p* < 0.05; Table 2).

**Figure 2:**
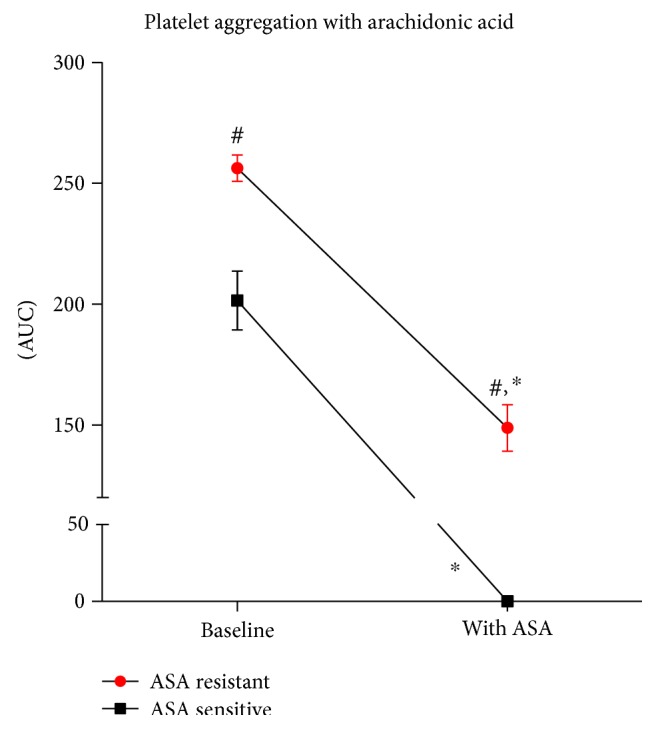
Platelet aggregation in response to arachidonic acid. Aggregations performed in platelet-rich plasma at baseline and after preincubation with ASA; AUC: area under curve of aggregation; ^∗^*p* < 0.001 versus baseline; ^#^*p* < 0.001 versus the control group.

The AS population presented no platelet aggregation in response to arachidonic acid after PRP was preincubated with a fixed dose of aspirin. Otherwise, in AR, the aggregation was only partially blocked (256.2 ± 5.2 versus 148.8 ± 9.3 AU, *p* < 0.001). Furthermore, the difference between study groups was observed not only after ASA incubation (148.8 ± 9.5 versus 0.08 ± 0.08 AU, *p* < 0.001) but also at baseline (AA-induced aggregation without ASA, 256.2 ± 5.2 versus 201.4 ± 12.0 AU, *p* < 0.001) (Figure 2).

### 3.3. Proteomic Analysis

**Figure 3:**
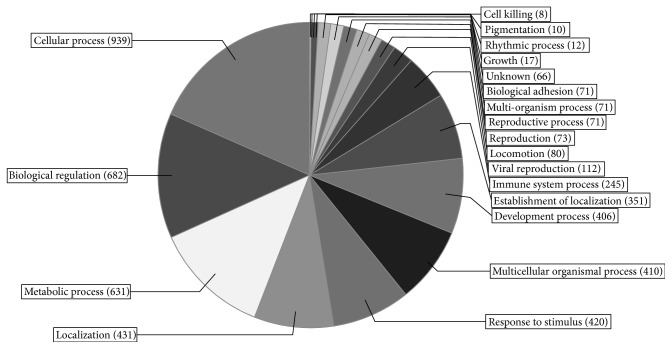
Platelet proteome organized according to involvement in biological processes of recognized proteins.

**Figure 4:**
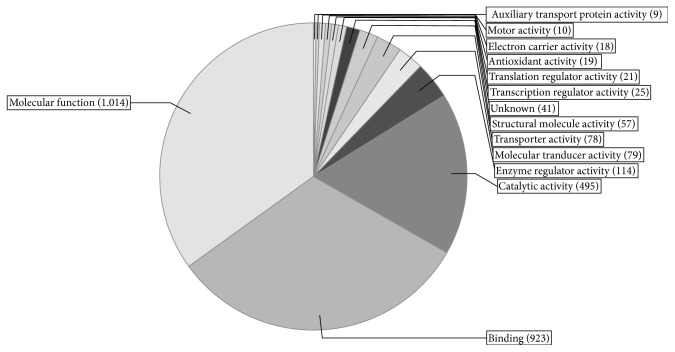
Platelet proteome organized according to molecular function of recognized proteins.

After preliminary analytical procedures and focusing on statistically significant proteins, we identified an average of 842 proteins per patient, based on an average of 7733 peptides. The obtained results demonstrated the reproducible sample preparation, enabling thorough quantitative analysis. In the next step, the identified proteins were divided according to molecular functions and their involvement in biological processes as shown in Figures 3 and 4. Subsequent quantitative analysis revealed differentiating proteins. The differential analysis of platelet proteome between study groups showed that the only discriminatory protein, affecting the response to aspirin, is carbonic anhydrase II. Exactly the same results were obtained in different settings, both in the collagen-induced aggregates and in the nonaggregated platelets (Table 3).

## 4. Discussion

This is the first study to demonstrate that carbonic anhydrase II (CA II) may be linked to human aspirin resistance (AR). The CA II protein (enzyme catalogue (EC) 4.2.1.1) in platelets has been described for the first time over 30 years ago [20] and first mentioned 60 years ago [21]. It catalyzes H^+^ and HCO_3_^−^ generation from CO_2_ and H_2_O contributing to pH changes [22]. Nevertheless, the exact role of CA II in the platelet pathophysiology remains not fully understood. A mutation in the CA II gene results in morphological and functional changes in mouse platelets [23].

The groups in our study were homogenous allowing to assume that the only discriminator is aspirin response. We used the LC/MS technique, which is much more reliable and investigator independent than the other ones, including the 2-dimensional electrophoresis [24, 25].

One of the explanation of the CA II, ASA interaction, observed in this study may be a hypothesis that by adjusting of the cytosolic pH [22], the CA II affects the acetylating of platelet cyclooxygenase by aspirin which could in turn determine their response to the ASA.

We have shown that platelets with greater CA II concentrations not only require lower arachidonic acid concentration to overcome ASA blockage but also produce more potent baseline aggregation. We postulate that platelet CA II increases platelet sensitivity to agonists like adrenaline, thrombin, and arachidonic acid. For this reason, humans with higher platelet CA II concentration and/or activity might be at higher risk of thromboembolic events. Hence, we postulate that the importance of CA in cardiovascular medicine seems to be still underestimated. Interestingly, numerous drugs commonly used in cardiovascular medicine (mainly diuretics) inhibit CA. The CA II activity can be stimulated by adrenaline [26]. Furthermore, adrenaline's potential to initiate aggregation is proportional to the platelet CA II activity and may by attenuated by the CA inhibitors like chlorthalidone [27]. Acetazolamide, a selective CA II inhibitor, decreases both basal- and adrenaline-induced platelet cytosolic chloride concentration and decreases thrombin sensitivity [28] similarly to another CA inhibitor (ethoxzolamide) [29]. Such effect may be also obtained by eliminating CO_2_ from the platelet environment [30]. What is more, the authors of a recent systemic review confirmed that thiazide-like diuretics have a superiority in reducing cardiovascular events over thiazide-type ones independently of lowering the blood pressure [31], which might be due to their activity against CA II and subsequent platelet aggregation [27].

Our results regarding sex distribution among AR population confirm the data published by other groups [32], and it should be verified if platelet CA II presents different sex-dependent activities. Lower red blood cells (RBC), hematocrite, and the hemoglobin levels in the AR population are accompanied by higher platelet CA II content. Anemia caused by iron deficiency has been demonstrated to be a risk factor for ischemic stroke due to reactive thrombocytosis, which might be a compensatory mechanism providing sufficient CA activity, normally substantially maintained by RBCs [33]. Platelet CA II might cooperate with the CA in RBCs in maintaining the acid-based blood balance. In our study, a thrombocytosis was not well marked, but increased CA II expression might induce differences in platelet function.

## 5. Conclusions

Carbonic anhydrase II (CA II) is a modulator of platelet function. Increased activity and/or concentration of CA II in platelets should be rated as a new independent risk factor for aspirin resistance and thus for thromboembolic events. There may be a need to use the drugs inhibiting CA in clinical setting more often nowadays, especially in patients with increased platelet activity/amount of CA II.

## Figures and Tables

**Table 1:** Baseline demographic characteristics and biochemical stratification of cardiovascular risk in subgroups separated according to the ASA response.

—	Aspirin resistant (AR) (mean ± SEM)	Aspirin sensitive (AS) (mean ± SEM)	*p*
*N*	*36–*	*25–*	
Women [%]	22 (61%)	5 (20%)	
Age [y]	30.28 ± 1.74	29.16 ± 1.81	0.41
BMI [kg/m^2^]	22.87 ± 0.61	24.11 ± 0.67	0.10
WBC [k/μl]	5.77 ± 0.22	5.66 ± 0.26	0.80
RBC [mln/*μ*l]	4.68 ± 0.08	4.94 ± 0.09	0.02
Hemoglobin [g/dl]	13.96 ± 0.25	14.74 ± 0.30	0.03
Hematocrit [%]	40.12 ± 0.64	42.12 ± 0.69	0.05
MCV [fl]	85.89 ± 0.51	86.10 ± 0.67	0.82
MCH [pg]	29.86 ± 0.20	30.09 ± 0.35	0.46
MCHC [g/dl]	34.77 ± 0.15	34.95 ± 0.22	0.54
PLT [k/μl]	236.72 ± 9.98	213.76 ± 9.32	0.12
PDW [fl]	13.79 ± 0.37	13.55 ± 0.46	0.50
Glucose [mg/dl]	83.39 ± 1.07	88.52 ± 2.06	0.03
Creatinine [mg/dl]	0.96 ± 0.02	1.11 ± 0.03	<0.001
eGFR [ml/min]	82.78 ± 1.51	79.76 ± 1.84	0.25
Uric acid [mg/dl]	7.25 ± 1.68	5.49 ± 0.22	0.18
hsCRP [mg/l]	1.77 ± 0.44	0.74 ± 0.18	0.06
Total cholesterol [mg/dl]	179.97 ± 6.16	190.52 ± 6.38	0.35
HDL [mg/dl]	54.25 ± 1.89	53.20 ± 1.87	0.89
LDL [mg/dl]	105.72 ± 5.62	115.96 ± 5.47	0.26
Triglycerides [mg/dl]	90.42 ± 7.97	106.84 ± 15.71	0.71
Sodium [mmol/l]	137.83 ± 0.27	138.16 ± 0.26	0.54
Potassium [mmol/l]	4.04 ± 0.04	4.18 ± 0.05	0.10

BMI: body mass index; WBC: white blood cells; RBC: red blood cells; MCV: mean (red blood) cell volume; MCH: mean corpuscular hemoglobin; MCHC: mean corpuscular hemoglobin concentration; PLT: platelets; PDW: platelet distribution width; eGFR: estimated glomerular filtration rate; hsCRP: high-sensitivity C-reactive protein; HDL: high-density lipoprotein; LDL: low-density lipoprotein.

**Table 2:** Comparison of prostanoids and markers of platelet activation in subgroups separated according to ASA response.

—	Decreased ASA response (mean ± SEM)	Normal ASA response (mean ± SEM)	*p*
*N*	*36–*	*25–*	*—*
PAI-1 [ng/ml]	3.11 ± 0.31	3.66 ± 0.32	0.04
Sel-P [ng/ml]	37.45 ± 2.10	40.26 ± 3.07	0.56
TxB_2_ [pg/ml]	1557.8 ± 410.7	826.2 ± 144.6	0.28
6-keto-PGF1*α* [pg/ml]	215.16 ± 20.77	187.98 ± 18.41	0.40

PAI-1: plasminogen activator inhibitor-1; Sel-P: P-selectin; TxB_2_: thromboxane B_2_, 6-keto-PGF1*α*: 6-keto prostaglandin F1*α*.

**Table 3:** Quantitative LC/MS analysis of proteomes of platelets sensitive versus resistant to ASA (only discriminatory proteins are specified).

Material	Protein	*q* value	Ratio	Fold change	Number of peptides	Protein name
Collagen-induced aggregation pellets	P00918	0.00213	0.67	1.49	18	*Carbonic anhydrase 2 OS = Homo sapiens*
Inactivated platelets	P00918	0.00106	0.69	1.44	19	*Carbonic anhydrase 2 OS = Homo sapiens*
